# S100a4 Is Secreted by Alternatively Activated Alveolar Macrophages and Promotes Activation of Lung Fibroblasts in Pulmonary Fibrosis

**DOI:** 10.3389/fimmu.2018.01216

**Published:** 2018-06-01

**Authors:** Wei Zhang, Shinji Ohno, Beatrix Steer, Stephan Klee, Claudia A. Staab-Weijnitz, Darcy Wagner, Mareike Lehmann, Tobias Stoeger, Melanie Königshoff, Heiko Adler

**Affiliations:** ^1^Comprehensive Pneumology Center, Research Unit Lung Repair and Regeneration, Helmholtz Zentrum München – Deutsches Forschungszentrum für Gesundheit und Umwelt (GmbH), Munich, Germany; ^2^University Hospital Grosshadern, Ludwig-Maximilians-Universität München, Munich, Germany; ^3^German Center for Lung Research (DZL) Giessen, Germany; ^4^Institute of Lung Biology and Disease, Comprehensive Pneumology Center, Helmholtz Zentrum München – Deutsches Forschungszentrum für Gesundheit und Umwelt (GmbH), Neuherberg, Germany; ^5^Division of Pulmonary Sciences and Critical Care Medicine, Department of Medicine, University of Colorado Denver, Aurora, CO, United States

**Keywords:** S100A4, alternatively activated macrophages, lung fibrosis, fibroblast activation, fibroblast proliferation, M2 macrophages

## Abstract

Idiopathic pulmonary fibrosis (IPF) is a devastating interstitial lung disease, characterized by damage of lung epithelial cells, excessive deposition of extracellular matrix in the lung interstitium, and enhanced activation and proliferation of fibroblasts. S100a4, also termed FSP-1 (fibroblast-specific protein-1), was previously considered as a marker of fibroblasts but recent findings in renal and liver fibrosis indicated that M2 macrophages are an important cellular source of S100a4. Thus, we hypothesized that also in pulmonary fibrosis, M2 macrophages produce and secrete S100a4, and that secreted S100a4 induces the proliferation and activation of fibroblasts. To prove this hypothesis, we comprehensively characterized two established mouse models of lung fibrosis: infection of IFN-γR^−/−^ mice with MHV-68 and intratracheal application of bleomycin to C57BL/6 mice. We further provide *in vitro* data using primary macrophages and fibroblasts to investigate the mechanism by which S100A4 exerts its effects. Finally, we inhibit S100a4 *in vivo* in the bleomycin-induced lung fibrosis model by treatment with niclosamide. Our data suggest that S100a4 is produced and secreted by M2 polarized alveolar macrophages and enhances the proliferation and activation of lung fibroblasts. Inhibition of S100a4 might represent a potential therapeutic strategy for pulmonary fibrosis.

## Introduction

Idiopathic pulmonary fibrosis (IPF) is the most devastating interstitial lung disease (ILD), more deadly than most cancers, and death usually ensues 2–5 years after diagnosis due to respiratory failure (1, 2). It is characterized by damage of lung epithelial cells, excessive deposition of extracellular matrix (ECM) in the lung interstitium, and enhanced activation and proliferation of fibroblasts, which ultimately leads to the distortion of normal lung architecture and loss of lung function (3). The underlying cause of IPF is not known (4) but recent studies imply that the interplay between genetic factors [mutations and polymorphisms (SNPs)] and environmental factors ultimately lead to IPF (3). Recently, the FDA approved Ofev (nintedanib) and Esbriet (pirfenidone) for the treatment of IPF patients (5–7). However, these therapies do not work for all patients and new medicines with fewer adverse effects are highly desirable.

S100a4, also termed FSP-1 (fibroblast-specific protein-1), was previously considered as a marker of fibroblasts in different organs undergoing tissue remodeling including kidney, lung, liver, and heart (4, 8). Upregulation of S100a4 was observed in fibrotic lung tissue of patients and attributed to an increase in FSP-1 expressing fibroblasts (9) or to an increased expression by SFTPC (pulmonary-associated surfactant protein C) positive alveolar epithelial cells (10). However, in the mouse model of kidney injury, S100a4 was found to be co-expressed with macrophage antigens Mac1, Mac2, and Mac3 as well as CD45 and CD68 (11). Similar findings were reported in renal and liver fibrosis (12–14). Thus, it is conceivable that also in lung fibrosis, macrophages could be an important cellular source of S100a4. Macrophages display various activation states, and the main activation phenotypes are classically activated (M1) and alternatively activated (M2) macrophages. The pro-inflammatory M1 macrophages are often associated with inflammation and tissue injury, whereas the anti-inflammatory M2 macrophages are associated with tissue repair and fibrosis by secreting pro-fibrotic factors (15, 16). Prior investigations have suggested that M2 alveolar macrophages (AMs), expressing high levels of Ym1/2, FIZZ1, and Arg1, play an important role in driving fibrogenesis (17).

Here, we hypothesized that also in pulmonary fibrosis, M2 macrophages produce and secrete S100a4, and that the secreted S100a4 induces proliferation and activation of fibroblasts. To prove this hypothesis, we comprehensively characterized two established mouse models of lung fibrosis: infection of IFN-γR^−/−^ mice with MHV-68 and intratracheal application of bleomycin to C57BL/6 mice. In addition, we provide *in vitro* data using primary macrophages and fibroblasts to investigate the mechanism by which S100a4 exerts its effect. Finally, we inhibit S100a4 *in vivo* with niclosamide in the bleomycin-induced lung fibrosis model.

Our data suggest that S100a4 is produced and secreted by M2 polarized AMs and enhances the proliferation and activation of lung fibroblasts. Inhibition of S100a4 might represent a potential therapeutic strategy for pulmonary fibrosis.

## Materials and Methods

### Preparation and Titration of MHV-68

Virus stocks were grown and quantified by plaque assay as previously described (18).

### *In Vivo* Experiments

C57BL/6 mice were purchased from Charles River Laboratories (Sulzfeld, Germany). IFN-γ-R^−/−^ mice on C57BL/6 background were originally obtained from the Jackson Laboratory (Bar Harbor, ME, USA) and subsequently bred and propagated under SPF conditions at the Helmholtz Zentrum München. Mice were housed in individually ventilated cages during the MHV-68 infection period. Mice (8–12 weeks old) were infected intranasally (i.n.) with 1 × 10^5^ plaque forming units of MHV-68 diluted in PBS in a total volume of 30 µl. Prior to i.n. infection, mice were anesthetized with ketamine–xylazine or with medetomidine–midazolam–fentanyl. At the predetermined time points, mice were sacrificed by cervical dislocation and subsequently, bronchoalveolar lavage (BAL) was performed and lung tissues were quartered and processed for the following experiments: the left lobe was inflated and fixed in 10% buffered formalin for histological and immunohistochemical examination, and the remaining lobes were stored at −80°C and used for RNA isolation for qRT-PCR to determine gene expression or for preparation of whole lung tissue protein extracts and western blot analysis.

To induce pulmonary fibrosis, anesthetized mice (8–12 weeks old) were intratracheally instilled through a MicroSprayer Aerosolizer 20G INTROCAN (Penn Century, Wyndmoor, PA, USA) with 50 µl bleomycin (Sigma-Aldrich, Taufkirchen, Germany) in PBS (2 U/kg) or as control, with PBS alone. Mice were analyzed 14 days after bleomycin instillation. To test lung function, mice were anesthetized with ketamine/xylazine, intratracheally intubated through a small incision of the trachea and connected to the flexiVent system (Scireq, Montreal, Canada). Subsequently, mice were sacrificed and lung lobes were snap frozen and stored at −80°C before mRNA isolation and protein determination, and one lobe was filled with 4% paraformaldehyde for histology. Paraffin-embedded sections were stained with hematoxylin and eosin (H&E). Staining intensity was quantified by ImageJ.

All animal experiments were in compliance with the German Animal Welfare Act (German Federal Law §8 Abs. 1 TierSchG), and the protocols were approved by the local Animal Care and Use Committee (District Government of Upper Bavaria; permit number 124-08, 154-13, and 130-14).

### Bronchoalveolar Lavage

Immediately after euthanasia, BAL was conducted via the introduction of a cannula into the trachea. A 1 ml aliquot of ice-cold Dulbecco’s phosphate buffered saline (DPBS) was flushed into the airway and gently aspirated *via* a syringe and the tracheal cannula. After the first BAL fluid (BALF) was collected, the BAL continued with seven times of 1.5 ml aliquots of PBS until an additional 7 ml of BALF was collected. The initial BALF was then centrifuged at 1,500 rpm for 5 min at 4°C, and the supernatant was collected and decanted into a new 1.5 ml microcentrifuge tube and stored at −80°C for biochemical measurements such as cytokine concentration. The remaining lavage fluid was pooled and centrifuged to remove the supernatant. The sedimented cells together with remnant cell pellets from the first lavage wash were subsequently resuspended in 1 ml PBS. Finally, the number of living cells was counted on a standard hemocytometer in the presence of 0.4% trypan blue (Sigma-Aldrich).

### Histopathology

After BAL harvesting, either the whole lung or the left lobe was inflated by 5 ml of 10% phosphate buffered formaldehyde solution (PFA) (AppliChem) and then gently removed and immersed in 10% PFA. An average of three mice per group at each experimental time point was used for histopathological analysis. After fixation for 24 h, dissected lung tissues were dehydrated through a series of solutions with increasing concentrations of ethanol and subsequently embedded in paraffin blocks. 3 µm thick adjacent sections were cut by the microtome (Carl Zeiss), so that all parts of the samples were represented on the slides. Prior to H&E staining, slides were baked at 60°C for 30 min. Subsequently, lung sections were prepared for histopathological staining by deparaffinization in xylene, and rehydration in a decreasing ethanol series (100, 90, 80, and 70%, respectively) and distilled water. Slides were then stained with H&E according to the manufacturer’s protocols to determine histopathological changes and fibrosis. Briefly, lung sections were incubated in Mayer’s Hemalaun solution (Carl Roth) for 8 min, rinsed quickly in 0.3% acid–alcohol solution, washed, and then transferred into 0.5% eosin G solution (Carl Roth) for 8 min. Sections were washed in tap water and dehydrated in a graded ethanol series and covered with Entellan (Millipore).

### Immunohistochemistry (IHC)

Serial lung tissue sections were processed as follows: slides were deparaffinized and incubated with 3% hydrogen peroxide (H_2_O_2_) (Spectrum Chemical Mfg. Corp.) in 100% methanol for 20 min at room temperature to quench endogenous peroxidase activity. Heat-induced antigen retrieval was performed with 0.05% citrate buffer pH 6.0 (Dako REAL Target Retrieval Solution) for 30 s at 125°C and 10 s at 90°C. Subsequently, blocking was performed with Roden Block M buffer (Biocare Medical, Zytomed) for 1 h at room temperature to avoid non-specific antibody binding. To identify myofibroblasts or macrophages and products of alternatively activated macrophages, primary antibodies used were against α-smooth muscle actin (α-SMA), arginase I, and S100a4, according to the manufacturer’s instructions. The slides were incubated with corresponding secondary antibodies for 30 min at room temperature. Vulcan Fast Red Chromogen Kit (Biocare Medical, Zytomed) was used to visualize the positive stained cells and hematoxylin was used as counterstaining for nuclei. Isotype controls were routinely applied. The primary antibodies used for IHC staining were mouse anti-α-SMA monoclonal antibody (Sigma-Aldrich) diluted at 1:200 in antibody diluent (Zytomed Systems), rabbit anti-arginase I polyclonal antibody (Santa Cruz Biotechnology Inc.) at 1:200 dilution and rabbit anti-S100a4 polyclonal antibody (Abcam) at 1:250 dilution. The secondary antibody applied was the rabbit-on-rodent alkaline phosphatase (AP) polymer (Biocare Medical, Zytomed) and mouse-on-mouse AP-polymer (Biocare Medical, Zytomed).

### Immunocytofluorescence Staining

For immunocytofluorescence staining of arginase I and S100a4, primary AMs were seeded on a 24-well plate containing sterile round glass coverslips at a density of 5 × 10^5^ cells/well and cultured in the absence or presence of IL-4 (20 ng/ml). At 48 h after the initiation of stimulation, adherent cells were washed with precooled PBS and fixed with cold methanol for 10 min at room temperature. After washing three times in PBS for 5 min, cells were covered with blocking solution (1% BSA in PBS) for 30 min at room temperature, and then incubated with a mixture of mouse anti-S100a4 monoclonal antibody (Abcam) at 1:300 dilution and rabbit anti-arginase I polyclonal antibody (Santa Cruz Biotechnology Inc.) at 1:250 dilution for 1 h at room temperature. This was followed by an exposure to a mixture of secondary antibodies, a goat anti-mouse IgG Alexa Fluor^®^ 488 secondary antibody (Thermo Scientific) and a goat anti-rabbit IgG Alexa Fluor^®^ 633 secondary antibody (Thermo Scientific), at 1:500 dilution, in the dark for 1 h. Stained cells were mounted with Mowiol which contains 4′,6-diamidino-2-phenylindole (DAPI) (Dako) to visualize the nuclei, and images were captured by a fluorescence microscope (LSM 700, Carl Zeiss). The appropriate irrelevant isotype-matched immunoglobulins were employed as negative controls.

For immunocytofluorescence staining of S100a4 and Mac3, lung sections were deparaffinized and hydrated using standard procedures. Sections were immersed into 3% H_2_O_2_ diluted in methanol for 20 min at room temperature to quench endogenous peroxidase. Antigen retrieval was performed using citrate buffer (pH6) and pressure cooker heating. Blocking was performed with 1% BSA for 1 h at room temperature. Subsequently, sections were incubated overnight at 4°C with a mixture of rabbit anti-S100a4 polyclonal antibody (Abcam) at 1:100 dilution and rat anti-mouse Mac3 monoclonal antibody (BD) at 1:50 dilution for 1 h at room temperature. After washing three times, immunofluorescence detection was performed using mixed secondary antibodies as follows: goat anti-rabbit Alexa Fluor^®^ 633 (Thermo Scientific) and goat anti-rat Alexa Fluor^®^ 488 (Thermo Scientific), both at 1:500 dilution, in the dark for 1 h. Slides were mounted with fluorescence mounting medium containing DAPI (DAKO) to visualize the nuclei, and images were captured by a fluorescence microscope (LSM 700, Carl Zeiss).

Immunofluorescent stainings of S100A4 and CD163 in tissue sections from human lungs were performed using a mouse monoclonal anti-CD163 antibody (Novus, NBP2-36494, 1:200) and a rabbit polyclonal anti-S100A4-antibody (abcam, ab41532, 1:100), essentially as described previously (19). Human lung tissue was obtained from the Comprehensive Pneumology Center cohort of the BioArchive CPC-M at the University Hospital Grosshadern of the Ludwig Maximilian University. Participants provided written informed consent to participate in this study, in accordance with approval by the local ethics committee of the LMU, Germany (Project 333-10, 455-12).

### RNA Isolation From Lung Tissue

Approximately, 20 mg frozen lung tissue was disrupted and completely homogenized in 350 µl TissueLyser LT buffer through the FastPrep-24 Lysator (MP Biomedicals). Total RNA was extracted and purified by utilizing the RNeasy Mini Kit (Qiagen) as per the manufacturer’s instruction. The concentration and purity of RNA samples were quantified by a Nanodrop ND-1000 spectrophotometer (Thermo Scientific). RNA was stored at −80°C for quantitative real-time RT-PCR.

### cDNA Synthesis

cDNA was generated from total RNA utilizing SuperScript RT III kit (Invitrogen) in a total volume of 20 µl. First-strand synthesis was performed in a total volume of 13.5 µl with 1 µg RNA, 1 µl 10 mM dNTP (NEB), 1 µl random hexamers (50 µM), and RNase-free distilled water. The mixture was incubated at 65°C for 5 min and then chilled on ice immediately for at least 1 min. Thereafter, the following reagents were added to the RNA/hexamers mixture: 4 µl 5 × first-strand buffer, 2 µl 0.1 mM DTT, and 0.5 µl of Superscript III (200 units). The obtained solution was incubated at 42°C for 1 h and reverse transcriptase was inactivated by incubating at 70°C for 10 min. cDNA was either stored at −20°C or subjected to quantitative real-time PCR analysis (diluted 1:10 with H_2_O before use).

### Quantitative Real-Time PCR

Quantitative real-time PCR (qPCR) was conducted using the ABI Prism 7300 real-time PCR System (Applied Biosystems, Life Technologies). Specific primers for the genes of interest were custom designed through the online PrimerBank database (https://pga.mgh.harvard.edu/primerbank/) and synthesized by Metabion (Martinsried, Germany). The housekeeping genes ribosomal protein l8 or β-actin were used to normalize for the input of loaded cDNA. 1 µl of cDNA was mixed with the appropriate 100 nmol/ml primers and 2 × SYBR Green Master Mix (Applied Biosystems, Life Technologies) in a total volume of 25 µl. The sequences of primers are given in Table 1. Each qPCR reaction was carried out in a 96-well plate in duplicate with the following program: 95°C for 10 min for initial denaturation, 40 cycles of amplification as follows: (1) denaturation at 95°C for 15 s, (2) annealing and elongation at 60°C for 1 min. Melting curve analysis was also done with a continuous temperature increasing from 60°C to 95°C with a rate of 0.1°C/s to assess the specificity of the amplification process. Relative gene expression levels were calculated using the comparative Ct (ΔΔCt) method.

**Table 1:** Primer sequences of genes of interest (GOI).

Target gene	Forward primer (5′–3′)	Reverse primer (5′–3′)
Actb	TCCATCATGAAGTGTGACGT	GAGCAATGATCTTGATCTTCAT
Arg1	GGAACCCAGAGAGAGCATGA	TTTTTCCAGCAGACCAGCTT
Tnf	CACCACGCTCTTCTGTCT	GGCTACAGGCTTGTCACTC
Ribosomal protein l8	AAGGCGCGGGTTCTGTTTT	GCTCTGTCCGCTTCTTGAATC
S100a4	TCAGCACTTCCTCTCTCTTGG	AACTTGTCACCCTCTTTGCC

### Protein Isolation and Quantification

Total protein was extracted from lung tissue with precooled RIPA buffer (10 mM Tris, 150 mM NaCl, 5 mM EDTA, 1% sodium deoxycholate, 1% Triton X-100, 1% SDS) supplemented freshly with one tablet complete protease inhibitor cocktail (Roche) per 10 ml. Samples were homogenized by the FastPrep-24 Lysator (MP Biomedicals) and placed on ice for 30 min with vigorous vortexing for every 10 min. All samples were centrifuged at 14,000 rpm for 15 min at 4°C, and supernatants were collected and decanted in new microcentrifuge tubes and stored at −80°C. For isolation of whole cell extracts, cells were washed with precooled PBS and lysed with cold RIPA buffer for 30 min on ice. Cell debris was pelleted at 14,000 rpm for 15 min at 4°C and the supernatant was harvested and stored at −80°C. Protein concentrations were measured by Bradford assay with coomassie protein assay reagent (Thermo Scientific) according to manufacturer’s instructions with BSA (Thermo Scientific) as standards (0, 25, 125, 250, 500, 750, 1,000, 1,500, and 2,000 µg/ml). Absorption at OD 595 was measured with a microplate absorbance reader (TECAN SUNRISE), and the BSA standard curve was applied to calculate concentrations of samples.

### Western Blot Analysis

Western blot was performed as follows: 25–50 µg total protein extract per sample was diluted in 4 × Laemmli Buffer (Biorad) supplemented with 5% β-mercaptoethanol and incubated for 5 min at 95°C. Samples were resolved in 5–15% sodium dodecyl sulfate polyacrylamide gels for 90 min at 120V in tris-glycine running buffer in an electrophoresis tank (Bio-Rad). The pre-stained full-range rainbow molecular weight marker (GE Healthcare, Life Science) was used to indicate the protein size. Proteins were transferred to the nitrocellulose membrane (GE Healthcare, Life Science) in blotting buffer at 300 mA for 60 min in a Hoefer TE22 Mini Tank (GE Healthcare, Life Science). The membrane was then soaked in 5% milk in TBS-T blocking buffer for 1 h at room temperature to prevent non-specific binding. Primary antibodies were diluted in 1% milk blocking buffer and incubated overnight at 4°C with agitation in a 50 ml falcon tube. After washing three times with TBS-T buffer, the membrane was incubated with the appropriate secondary antibody for 1 h at room temperature. The membrane was washed three times with TBS-T buffer, and the resulting signals were visualized and captured with Pierce ECL western blotting substrate (Thermo Scientific) and Bio-Rad imaging system (Thermo Scientific). If necessary, initial antibodies could be stripped with Restore Stripping Solution (Thermo Scientific) for 8–15 min at room temperature. The nitrocellulose membrane was then washed with TBS-T, blocked with 5% milk in TBS-T, and reprobed with other antibodies as described above. The antibodies and dilutions employed are shown in Table 2.

**Table 2:** Antibodies utilized in western blot assays.

Name	Dilution	Company
Rabbit polyclonal anti-S100a4 antibody	1:1,000	Abcam, ab27957
Mouse monoclonal anti-S100a4 antibody	1:1,000	Abcam, ab93283
Rabbit polyclonal anti-arginase I antibody	1:1,000	Santa Cruz, sc-20150
Rabbit polyclonal anti-GAPDH antibody	1:1,000	Abcam, ab37168
Rabbit polyclonal anti-STAT6 antibody	1:1,000	Cell Signaling, #9362
Rabbit polyclonal anti-pSTAT6 antibody	1:1,000	Cell Signaling, #9361
Mouse monoclonal anti-β-actin-HRP-conjugated antibody	1:50,000	Sigma-Aldrich, A3854
HRP-conjugated anti-mouse IgG secondary antibody	1:5,000	GE Health care, 9597364
HRP-conjugated anti-rabbit IgG	1:5,000	GE Health care, 356938

### Determination of S100a4 by ELISA

Secreted S100a4 in cell culture supernatants or in BAL fluid was measured using a published sandwich ELISA (13). Briefly, 96-well NUNC MaxiSorp microplates (NUNCTM, Thermo Scientific) were coated with 1 µg/ml mouse anti-S100a4 monoclonal antibody (Abcam) at 4°C overnight. After blocking with assay buffer (PBS + 5% BSA), samples were added and incubated for 2 h at 37°C, followed by incubation with 1 µg/ml rabbit polyclonal anti-S100a4 antibody (Abcam) at room temperature with continuous shaking (700 rpm). Next, a secondary HRP-conjugated anti-rabbit IgG (GE Healthcare, Life Science) was applied and proteins were detected by TMB substrate.

### Isolation of Mouse Primary Resident AMs

To isolate lung AMs, C57BL/6 wild-type mice were anesthetized by injection of xylazine and ketamine intraperitoneally and killed by exsanguination. The trachea was exposed, cannulated, and the lungs were serially washed with 1 ml sterile PBS 10 times as described previously to harvest lavage fluid. Cell pellets were obtained by centrifugation at 1,500 rpm for 10 min at 4°C, and 5 × 10^5^ cells per well were seeded in 24-well plate in complete RPMI-1640 medium (Gibco) and incubated at 37°C and 5% CO_2_ atmosphere. Cells were allowed to adhere for 60–90 min and then non-adherent cells were removed by washing twice with PBS.

### Isolation and Cell Culture of Mouse Primary Lung Fibroblasts

C57BL/6 mice were euthanatized by exsanguination as described above. 15 ml cold PBS was perfused smoothly into the right heart ventricle until the lung got cleared of blood. The whole lung was removed and rinsed in pre-warmed DMEM/F-12 (Gibco) medium supplemented with 1% penicillin/streptomycin (Gibco), 1% HEPES buffer (Gibco), and 15% FBS (PAA). The lung was diced into 1–2 mm pieces and digested with 0.1 mg/ml collagenase A (Roche) at 37°C for 2 h. Digested tissue was then minced using a 100 µm cell strainer (BD, Biosciences). After washing and centrifuging, cells were resuspended in complete DMEM/F-12 medium and incubated at 37°C with 5% CO_2_. The culture medium was changed every 2–3 days to remove unattached cells. After reaching 80–90% confluence, cells were detached by 0.25% trypsin (Gibco), split at 1:4 and applied to experiments at passages not higher than three.

### Macrophage Activation Experiments

Isolated primary AMs were cultured in 24-well plates (5 × 10^5^ cells/well) in RPMI-1640 medium (Gibco) supplemented with 1% penicillin/streptomycin (Gibco) and 10% FBS (PAA) overnight. The cells were stimulated with LPS (100 ng/ml, Sigma) and/or IFN-γ (20 ng/ml, Immuno Tools) to produce M1 macrophages, or with IL-4 (20 ng/ml, Immuno Tools) to induce M2 macrophage polarization. The application of a 72-h time course (6, 24, 48, and 72 h) and increasing doses of IL-4 (10, 20, 50, 100, and 200 ng/ml) allowed for the accurate analysis of gene expression profiles and cytokine release without medium change or repeated administration of stimuli. Untreated macrophages incubated in culture medium served as controls. For the analysis of cytokines, supernatants were collected and subjected to ELISA for measurement of S100a4 as described above. Adherent cells were washed with ice-cold PBS and then harvested for total RNA or protein isolation.

### Cell Proliferation Assay

Cell proliferation was evaluated after different treatments by utilizing the cell proliferation kit II (XTT) (Roche) according to the manufacturer’s instructions. Briefly, primary lung fibroblast cells were plated into 96-well plates (3 × 10^3^ cells/well) in complete DMEM/F-12 medium (Gibco) and allowed to accommodate overnight before quiescing by replacing with serum-free medium for a further 12 h. Subsequently, cells were incubated in 2% FBS DMEM/F-12 medium with or without recombinant S100a4 protein (2 µg/ml) or together with the anti-S100a4 antibody (3 µg/ml) (R&D Systems) for 72 h. Prior to harvesting, cells were treated with XTT labeling mixture for 4 h, and the absorbance was quantified at 450 nm with a reference wave length at 650 nm by using a microplate absorbance reader (TECAN SUNRISE). To verify whether the inhibition of S100a4 in M2 macrophages influences the pro-proliferative effect, M2 macrophage conditioned medium was incubated with S100a4 antibody (3 µg/ml) for 1 h at room temperature, and then applied on pre-starved primary lung fibroblast cells. Additionally, the conditioned medium from S100a4-siRNA transfected M2 macrophages was also applied on pre-starved cells. After 48 h, proliferation was evaluated by cell proliferation kit II (XTT) as mentioned above.

### Wound Healing Assay

Cell migration was determined by wound healing assay. Initially, 5 × 10^4^ cells were seeded into a 24-well plate and allowed to adhere overnight until reaching about 70% confluence. Cell monolayers were inflicted by manually scraping with a 200 µl pipette tip and cell debris was removed by washing with PBS. Subsequently, cells were treated with the recombinant S100a4 protein (2 µg/ml) or that pre-incubated with the anti-S100a4 antibody (3 µg/ml) in culture medium supplemented with 2% FBS. Microphotographs were taken at 0 and 24 h after treatments with an Axiovert microscope (Carl Zeiss). The wound areas were subsequently measured and each wound healing assay was conducted in triplicates. Finally, the cells were harvested for further analysis.

### Transfection of Primary AMs With siRNA

For specific knockdown of the S100a4 gene in primary AMs, a set of three siRNAs as well as a non-targeting negative control was purchased from Riboxx (Radebeul, Germany) (NM_011311.2). Prior to the experiments, the siRNA powder was reconstituted with RNase-free water in order to obtain the working solution with a final concentration of 600 nM. Primary AMs were prepared as described above and seeded in 24-well plate at a density of 150,000 cells per well approximately 24 h before transfection. Cells were transfected in duplicates as follows: 3 µl riboxx^®^FECT transfection reagent diluted in 37 µl Opti-MEM (Gibco) were added to the 20 µl siRNA in 40 µl Opti-MEM. The reaction mixtures were incubated at room temperature for 15 min. In the meantime, the cell culture medium was aspirated and replaced with 500 µl fresh complete medium. Then, the mixture was added to each designed well to a final siRNA concentration of 20 nM/well. After transfection for 72 h, supernatants were harvested for further experiments, and cells were harvested for RNA extraction and subsequent quantitative RT-PCR analysis.

### Cytotoxicity Assay

The inhibitors of S100a4, calcimycin, and niclosamide (2′,5-dichloro-4′-nitrosalicylanilide), were purchased from Sigma and were solubilized in dimethyl sulfoxide (DMSO) for *in vitro* experiments. To exclude adverse effects caused by DMSO, control cells were treated with the equal amount of solvent. Analysis of cell cytotoxicity was performed with the cell proliferation kit II (XTT) (Roche) according to the manufacturer’s instructions. Briefly, cells were seeded at 1 × 10^4^ cells per well in a 96-well plate and allowed to accommodate overnight. The cells were exposed to a series of different concentrations of calcimycin or niclosamide for 24 h. Subsequently, cells were treated with XTT labeling mixture for 4 h, and the absorbance was quantified at 450 nm with a reference wave length at 650 nm by using a microplate absorbance reader (TECAN SUNRISE). Cell viability was determined by dividing the absorbance ratio of drug-treated cells by the ratio obtained from untreated cells which was defined as 100% cell viability.

### Pharmacologic Inhibition of S100a4 Expression *In Vivo*

Mice were treated with niclosamide (Sigma) as described (20). Briefly, niclosamide (20 mg/kg) as suspension in 10% Cremophor EL (BASF, Ludwigshafen, Germany) and 0.9% NaCl solution was administered i.p. in a volume of 150 µl once daily from day 7 to day 13 after bleomycin instillation. Control mice were treated with the appropriate volume of solvent solution (10% Cremophor EL and 0.9% NaCl).

### Statistical Analysis

All statistics and calculations were conducted with GraphPad Prism version 6.0. Values are shown as mean ± SD of at least three animals or three individual samples in each group if not otherwise indicated. The comparison of two groups was examined by unpaired Student’s *t*-test, and comparisons between several experimental groups were performed by analysis of variance. All significance tests were two-tailed and P values were expressed as follows: 0.05 > *p* > 0.01 as *; 0.01 > *p* > 0.001 as **; *p* < 0.001 as ***; *p* < 0.0001 as ****.

## Results

### Expression of S100a4 in Two Different Animal Models of IPF

**Figure 1:**
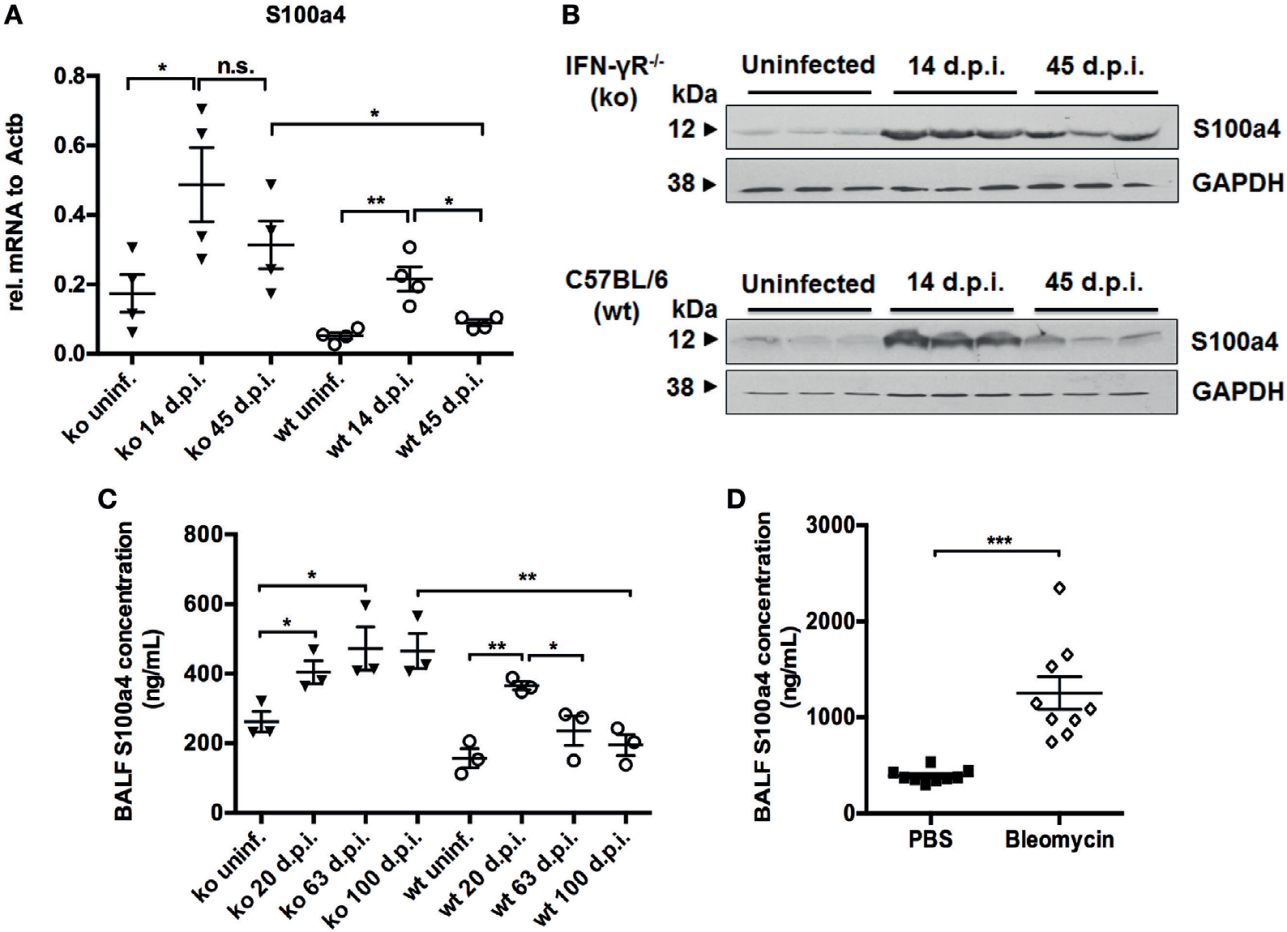
Analysis of S100a4 expression in two different mouse models of pulmonary fibrosis. **(A)** RNA was isolated from uninfected and from MHV-68-infected IFN-γR^−/−^ (ko) and C57BL/6 wild-type (wt) mice at days 14 and 45 after infection. S100a4 expression was analyzed by qRT-PCR, normalized to the expression of β-actin, and depicted as relative fold changes. Results are derived from three mice per group and shown as mean ± SD. **(B)** Lung homogenates from uninfected mice and MHV-68-infected mice at the indicated time points were subjected to western blot analysis for the S100a4 protein (three mice per group). Blots were either incubated with an anti-S100a4-antibody or an anti-GAPDH antibody as loading control. **(C)** S100a4 protein was measured in bronchoalveolar lavage (BAL) fluids from uninfected or MHV-68-infected IFN-γR^−/−^ (ko) and C57BL/6 mice (wt) at days 20, 63, and 100 after infection. Each symbol represents a mouse. Results are derived from three mice per group and shown as mean ± SD. Unpaired *t*-test was performed for statistical analysis (* denotes *p* < 0.05; ** denotes *p* < 0.01; n.s. denotes non-significance). **(D)** Protein levels of S100a4 were measured in BAL fluid from PBS or bleomycin-treated C57BL/6 mice at 14 days after instillation. Each symbol represents a mouse. Results are derived from nine mice per group and shown as mean ± SD. Unpaired *t*-test was performed for statistical analysis (*** denotes *p* < 0.001).

First, we analyzed the expression of S100a4 in the MHV-68 mouse model of IPF by qRT-PCR. S100a4 was highly expressed at day 14 p.i. (acute inflammation phase) in both IFN-γR^−/−^ and wild-type (C57BL/6) mice. However, while it declined to baseline in wild-type mice, it remained high in IFN-γR^−/−^ mice at day 45 p.i. (fibrotic phase) (Figure 1A). This could also be shown at the protein level (Figure 1B). Since it has been reported that S100a4 could also be secreted (21), an ELISA was performed to analyze S100a4 protein in the BAL fluid from uninfected and MHV-68-infected IFN-γR^−/−^ and wild-type mice. The level of soluble S100a4 protein was elevated during the pulmonary inflammation (day 20 p.i.) in both strains of mice, and remained high during the fibrotic phase (>day 60 p.i.) in IFN-γR^−/−^ mice, while decreasing in wild-type mice (Figure 1C). Finally, the amount of S100a4 protein was also quantified in BAL fluid obtained from PBS- or bleomycin-treated C57BL/6 mice at 14 days after instillation, reflecting the fibrotic phase in this model (Figure 1D). An elevated expression level of S100a4 was observed in the bleomycin-treated mice. Consistent with previous results (22), these data indicate that a significantly increased level of S100a4 protein in the lung is a common phenomenon during fibrogenesis, independent of the experimental mouse model.

### IHC Localizes S100a4 to AMs in Fibrotic Mouse Lungs

**Figure 2:**
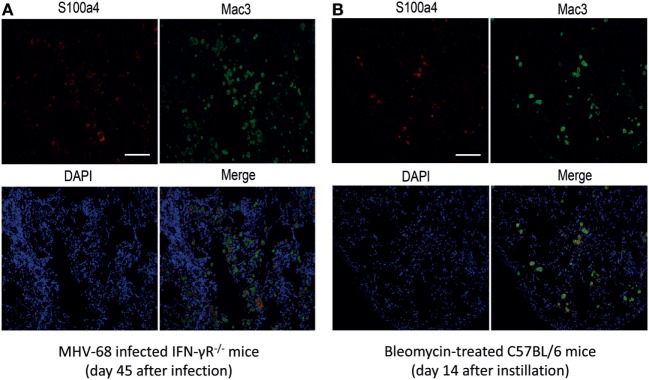
Co-localization of S100a4 with Mac3^+^ macrophages in tissue sections from fibrotic mice. **(A)** MHV-68-infected IFN-γR^−/−^ mice at 45 days after infection. **(B)** Bleomycin-treated C57BL/6 mice at 14 days after instillation. Green fluorescence: Mac3 antigen; red fluorescence: S100a4 antigen; blue fluorescence: diamidino-2-phenylindole-stained nuclei. In the merged image, yellow fluorescence indicates co-localization of S100a4 antigen and alveolar macrophages. All photomicrographs were taken at 200× magnification. Scale bar 50 µm.

In order to characterize the origin of S100a4 in the fibrotic lung tissue, immunohistochemical co-staining for S100a4 and the macrophage marker Mac3 was performed on lung sections of MHV-68 infected IFN-γR^−/−^ mice and bleomycin-treated mice. As shown in Figures 2A,B, there was a strong co-staining of Mac3 and S100a4, indicating that S100a4 is produced by macrophages present in the fibrotic lung. Since previous studies suggested that AMs driving the fibrotic process are activated by an alternative pathway (17), we hypothesized that S100a4 was secreted by alternatively activated macrophages. To confirm this, immunohistochemical staining of S100a4 and Arg1 on consecutive lung sections was performed which demonstrated that S100a4 positive cells matched with Arg1 positive cells (Figures S1A,B in Supplementary Material).

### IHC Localizes S100A4 to Macrophages in Human Lungs

**Figure 3:**
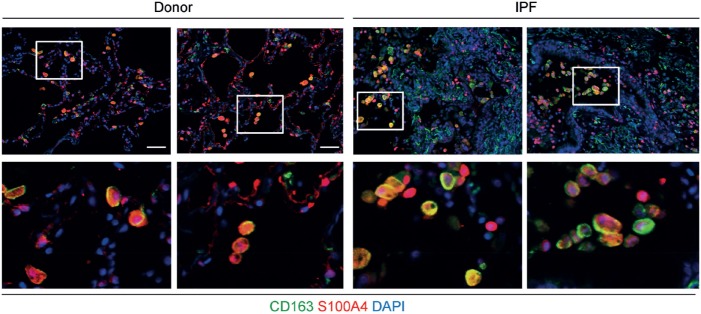
S100A4 is expressed by CD163+ macrophages in tissue sections from human lungs. Representative immunofluorescent stainings of paraffin sections from donor (left-hand panels) and idiopathic pulmonary fibrosis (IPF) tissue (right-hand panels) including higher magnification inserts in the bottom row. S100A4 is shown in red, the macrophage marker CD163 in green, and diamidino-2-phenylindole (DAPI) in blue. Scale bar 50 µm.

Next, we wanted to test whether S100A4 is also expressed by macrophages in the human lung. As a proof of concept, immunofluorescent co-staining for S100a4 and the macrophage marker CD163 was performed in lung sections. Macrophages expressing S100A4 were detected in lung sections of both, a control and an IPF patient (Figure 3). Consistent with these findings, S100A4 was also found to be present in BAL fluids of both control and IPF patients (data not shown). Clearly, for a quantitative analysis, a lot more human samples need to be analyzed.

### Analysis of S100a4 Expression in AMs Isolated From Control or Fibrotic Mice

To confirm that S100a4 is expressed by alternatively activated AMs, qRT-PCR was performed on freshly isolated AMs from uninfected and MHV-68-infected IFN-γR^−/−^ mice when lung fibrosis was well established (days 45 and 90 p.i.). Significant increases of S100a4 (fivefold) and Arg1, as well as a decrease of TNF-α, a typical marker of classically activated macrophages, were found in AMs derived from virus-infected mice, when compared with macrophages derived from uninfected mice (Figure S2A in Supplementary Material). Furthermore, also AMs isolated from bleomycin-treated mice highly expressed S100a4 and Arg1 but only weakly expressed TNF-α (Figure S2B in Supplementary Material). These findings support the hypothesis that S100a4 originates from alternatively activated macrophages during lung fibrosis.

### Analysis of S100a4 Gene Expression in Polarized AMs

Alveolar macrophages can be polarized into M1 and M2 phenotypes *in vitro* (23). Thus, we generated M1 and M2 macrophages, isolated total RNA, and performed qRT-PCR. The expression profile of *S100a4* in polarized AMs matched the expression pattern of *Arg1*. Both *S100a4* and *Arg1* were significantly elevated in M2 polarized AMs, when compared to the control M0 and M1 polarized macrophages (Figure S3 in Supplementary Material). Following the mRNA profile, we also investigated protein expression of S100a4 during alveolar macrophage polarization. Cells were treated with LPS or IFNγ and IL-4 or IL-13 for various times. The effects of IL-13 on activation of macrophages are similar to IL-4 (24), and both of them can induce the phosphorylation of STAT6 (25). Thus, phosphorylated STAT6 is used as a marker of M2 polarization. As expected, S100a4 protein was dectected after IL-4 and IL-13 induced M2 polarization but not in M0 or M1 polarized macrophages (Figures S4A,B in Supplementary Material). To confirm that S100a4 is secreted by M2 polarized macrophages, we performed ELISA tests on supernatants of IL-4-treated primary AMs from both IFN-γR^−/−^ and wild-type mice. The soluble S100a4 in the supernatant from IL-4-treated macrophages increased five to seven times compared with untreated cells (Figure S4C in Supplementary Material). Significantly, higher S100a4 production was found in M2 polarized AMs from IFN-γR^−/−^ mice compared to wild-type mice, which indicated that macrophages from IFN-γR^−/−^ mice possess an enhanced capacity for IL-4 stimulation, which is also consistent with our *in vivo* data (Figures 1A–C). Double immunofluorescence staining for S100a4 and Arg1 on M2-polarized macrophages showed co-localization of S100a4 and Arg1 (Figure S5 in Supplementary Material). Hence, the expression of S100a4 was substantiated by qRT-PCR, western blot, ELISA, and double immunofluorescence staining analysis, providing strong evidence that S100a4 is produced by M2 polarized AMs.

### Effect of S100a4 on the Activation, Proliferation, and Migration of Primary Lung Fibroblasts

**Figure 4:**
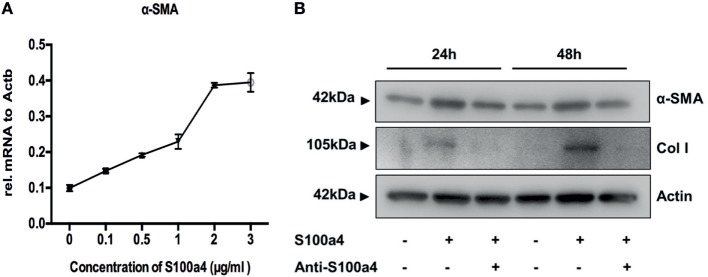
S100a4 promotes activation of lung fibroblasts. **(A)** Primary lung fibroblasts were treated with various concentrations of recombinant S100a4 (0–3 µg/ml) for 24 h, and expression of α-SMA was assessed by qRT-PCR. Results are mean ± SD of duplicate samples from one experiment. **(B)** Cells were treated with 2 µg/ml recombinant S100a4 or with recombinant S100a4 in the presence of a S100a4 neutralizing antibody for 24 and 48 h, respectively. Cells were harvested and analyzed for expression of α-SMA and collagen I by western blot. S100a4 neutralization eliminated activation of lung fibroblasts. Results are representative of three independent experiments with similar results.

**Figure 5:**
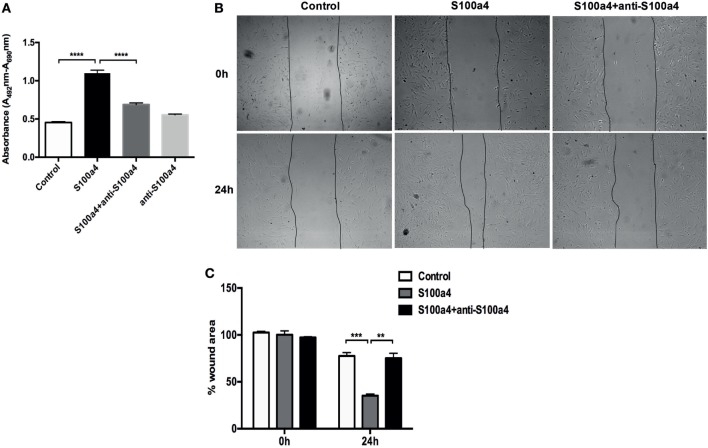
S100a4 accelerates lung fibroblast proliferation and migration. **(A)** Primary lung fibroblasts were treated with 2 µg/ml recombinant S100a4 or with recombinant S100a4 in the presence of a S100a4 neutralizing antibody or with antibody alone for 72 h. Cell proliferation was analyzed using the XTT kit. Results are representative of three independent experiments with similar results. Shown are mean ± SD of five replicates from one experiment. Unpaired t-test was performed for statistical analysis (**** denotes *p* < 0.0001). **(B)** Direct migration of primary lung fibroblasts in the presence of 2 µg/ml recombinant S100a4 or recombinant S100a4 in the presence of the S100a4 neutralizing antibody was analyzed by wound healing assay. Wound closure was determined 24 h after scratching. Representative phase-contrast pictures of the cells at 0 (immediately after the scratch) and 24 h after the scratch are shown. The assay was performed three times: one representative experiment is presented. **(C)** For quantification, the wound area was measured using ImageJ and normalized to control at 0 h. Results are representative of three independent experiments with similar results. Shown are mean ± SD of triplicate samples from one experiment. The effect of stimulation by S100a4 was statistically significant in comparison to the control sample, as evaluated by the unpaired *t*-test (** denotes *p* < 0.01; *** denotes *p* < 0.001).

M2 macrophages secrete Th2 cytokines, thereby promoting fibrogenesis *via* enhancing collagen deposition, angiogenesis, and fibroproliferation (26). Thus, S100a4 might serve as a cytokine-like factor indirectly promoting the pathogenesis of lung fibrosis. To investigate the influence of extracellular S100a4 on lung fibroblasts, primary mouse lung fibroblasts isolated from wild-type mice were treated with recombinant S100a4 for 24 h. As shown in Figure 4A, 0.1–3 µg/ml S100a4-induced significant expression of alpha-smooth muscle actin (α-SMA, a marker for myofibroblasts) in a concentration-dependent manner. When primary mouse lung fibroblasts were cultured in the presence of recombinant S100a4 protein, expression levels of α-SMA and collagen I were elevated 24 and 48 h after exposure, when compared to control cells (Figure 4B). This effect was blocked by neutralization of S100a4 with the specific antibody. These results indicate that S100a4 promotes activation of lung fibroblasts. Similarly, 72 h after stimulation, S100a4 significantly accelerated proliferation of lung fibroblasts when compared to control or antibody treated cells (Figure 5A). In fibrotic diseases, fibroblasts migrate to the wound site and participate in the construction of scar tissue. Hence, we performed a wound healing assay to investigate the influence of S100a4 on the migration of lung fibroblasts. Enhanced cell migration ability was observed in pulmonary fibroblasts after treatment with recombinant S100a4 which was significantly reduced by blockade with S100a4 neutralizing antibody (Figures 5B,C).

### Reduction of S100a4 Secretion by M2 Macrophages Diminishes Its Proliferative Effect on Primary Lung Fibroblasts

**Figure 6:**
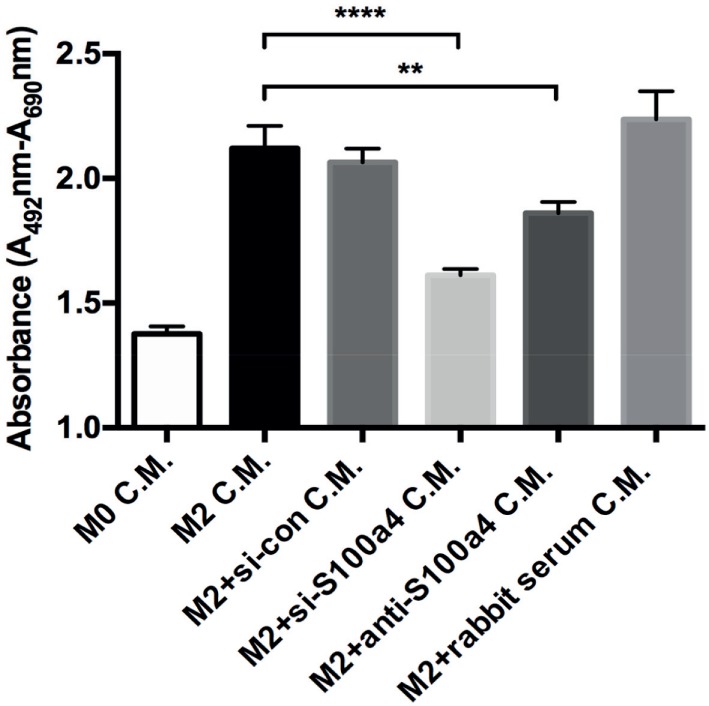
Effect of conditioned medium on lung fibroblast proliferation. Primary lung fibroblasts were treated with conditioned medium (C.M.) from M0, M2, and M2 macrophages transfected with scrambled or S100a4-specific siRNA. In addition, specific S100a4 antibody or isotype control rabbit serum pre-treated M2 conditioned medium were used to stimulate lung fibroblasts. Cell proliferation was analyzed by using XTT kit after 48 h of treatments. Results are representative of two independent experiments with similar results. Shown are mean ± SD of five replicates from one experiment. Unpaired *t*-test was performed for statistical analysis (** denotes *p* < 0.01; ****denotes *p* < 0.0001).

In order to investigate the role of endogenous S100a4 produced by M2 macrophages on primary lung fibroblasts with respect to proliferation and myofibroblast differentiation, AMs were isolated and *ex vivo* polarized into M2 macrophages by IL-4 treatment in the presence of anti-S100a4 siRNA or control siRNA. 72 h later, the supernatant was harvested and transferred to primary lung fibroblasts. Subsequently, the proliferation of the primary lung fibroblasts was analyzed (Figure S6A in Supplementary Material). S100a4 mRNA was efficiently downregulated by transient transfection with S100a4 specific siRNAs, when compared to transfection with control siRNA or to un-transfected M2 macrophages (Figure S6B in Supplementary Material). Furthermore, the amount of S100a4 protein in the supernatants of M2 macrophages was also efficiently downregulated (Figure S6C in Supplementary Material). Yet, inhibition of S100a4 during M2 polarization did not interfere with the expression of Arg1, which suggests that S100a4 is not involved in the polarization of macrophages (Figure S6D in Supplementary Material). To investigate the role of S100a4 produced by M2 macrophages on primary lung fibroblast proliferation, conditioned supernatants from control M2 macrophages or anti-S100a4 siRNA-transfected M2 macrophages were transferred to primary lung fibroblast cultures, and the effect on proliferation was evaluated after 24 h. Conditioned medium from anti-S100a4 siRNA-transfected M2 macrophages caused significantly less proliferation of lung fibroblasts when compared to conditioned medium from control macrophages (Figure 6). In addition, S100a4 neutralizing antibody also reduced supernatant-driven proliferation, while addition of control rabbit serum had no effect (Figure 6). These observations suggested that S100a4 is one of the soluble factors produced by M2 polarized AMs which are able to enhance the proliferation of fibroblasts.

### Pharmacologic Inhibition of S100a4 Expression

**Figure 7:**
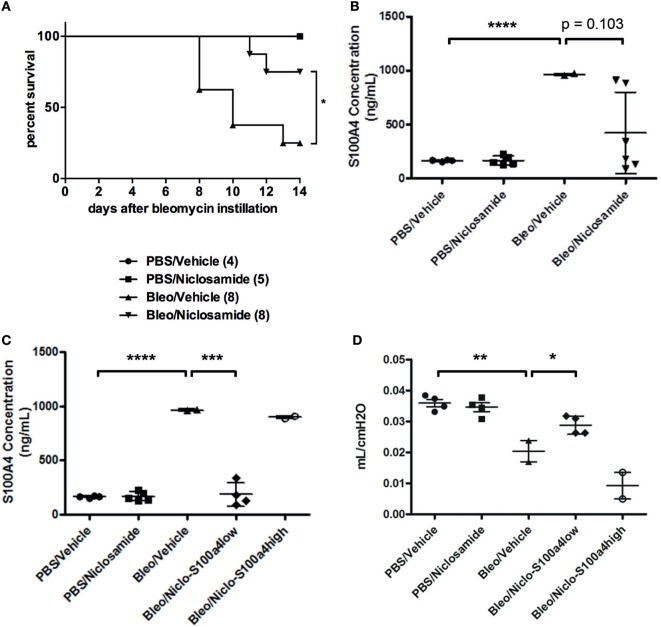
Niclosamide treatment in vivo. **(A)** Treatment with niclosamide improves survival of bleomycin-instilled mice. Mice were intratracheally treated with bleomycin, or as a control, with PBS. Beginning at day 7 after bleomycin instillation, mice were treated once daily with niclosamide (20 mg/kg) or vehicle. Mice were monitored daily for signs of disease, and any mice that appeared moribund were sacrificed. Treatment with niclosamide significantly improved the survival of bleomycin-instilled mice [*p = 0.031; Log-rank (Mantel–Cox) test]. Numbers in brackets indicate the total number of mice per group. **(B,C)** Treatment with niclosamide reduces S100a4 protein in the BAL fluid. The surviving mice depicted in panel **(A)** were subjected to BAL. The protein concentration of S100a4 in the BAL fluids was determined by ELISA. Each symbol represents a mouse. Results are shown as mean ± SD. Unpaired *t*-test was performed for statistical analysis (***p < 0.001; ****p < 0.0001). **(D)** Treatment with niclosamide improves lung function. The surviving mice depicted in panel **(A)** were subjected to a lung function test, and lung compliance was determined. Each symbol represents a mouse. Results are shown as mean ± SD. Unpaired *t*-test was performed for statistical analysis (*p < 0.05; **p < 0.01).

**Figure 8:**
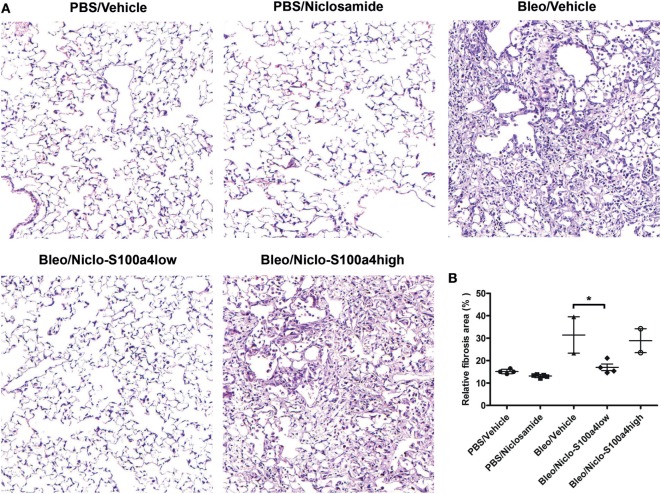
Treatment with niclosamide reduces the histological evidence of fibrosis. Lung tissue of the surviving mice depicted in Figure 7 was subjected to histological analysis. **(A)** Representative images of H/E-stained lung sections. **(B)** Quantitative analysis of all HE-stained sections. Each symbol represents a mouse. For each mouse, 3–5 sections were scanned using ImageJ. Results are shown as mean ± SD. Unpaired *t*-test was performed for statistical analysis (* denotes *p* < 0.05).

Previous studies reported that calcimycin and niclosamide, as transcriptional inhibitors of S100a4, can block S100a4 expression in colon cancer cells, thereby affecting metastasis (20, 27). To analyze the inhibitory potential of calcimycin and niclosamide on the expression of S100a4 in M2-polarized primary AMs, calcimycin, and niclosamide were applied to primary AMs during M2 polarization. Both compounds significantly reduced the S100a4 mRNA expression level but not the Arg1 expression (Figures S7A,B in Supplementary Material). Given the ability of calcimycin and niclosamide to inhibit S100a4 expression in primary AMs *in vitro*, we asked whether inhibition of S100a4 *in vivo* could attenuate the development of pulmonary fibrosis. We selected niclosamide, since it had been successfully applied before in mouse models of colon cancer (20). To induce lung fibrosis, mice were intratracheally treated with bleomycin, or as a control, with PBS. From day 7 after bleomycin instillation on, mice were treated daily with niclosamide or vehicle and monitored daily for signs of disease, and mice that appeared moribund were sacrificed. Treatment with niclosamide significantly improved the survival of bleomycin-instilled mice (Figure 7A). While in the group of vehicle-treated mice, 6 out of 8 animals had to be sacrificed until day 14, only 2 animals had to be sacrificed in the niclosamide-treated group. At day 14 after bleomycin instillation, the surviving mice were analyzed for S100a4 protein concentrations in the BAL fluids (Figure 7B). Consistent with our *in vitro* data, treatment with niclosamide reduced the levels of S100a4, when compared to vehicle treatment. However, the reduction did not reach statistical significance (*p* = 0.103), since it was observed in only 4 out of 6 niclosamide-treated mice. Obviously, in two mice, a reduction of S100a4 was not achieved by treatment with niclosamide. When we stratified the mice according to the S100a4 concentration in the BAL fluid (S100a4low versus S100a4high), the difference between the vehicle-treated mice and the niclosamide-treated, S100a4low mice became highly significant (Figure 7C). Second, lung function was determined (Figure 7D). Consistent with the BAL S100a4 concentrations, treatment of bleomycin-instilled mice with niclosamide significantly improved lung compliance in the four S100a4low mice but not in the two S100a4high mice, when compared to vehicle-treated mice, restoring lung function close to the level observed in mice instilled with PBS. Finally, lung tissue was histologically analyzed by staining with hematoxylin/eosin (Figure 8). As expected, no fibrosis was observed in control animals instilled with PBS, while strong evidence of fibrosis was noted in bleomycin-instilled animals treated with vehicle. Importantly, in bleomycin-instilled animals treated with niclosamide, fibrosis was significantly attenuated in the four S100a4low mice but not in the two S100a4high mice.

## Discussion

S100a4 was originally considered as a protein specifically expressed by fibroblasts. However, it has recently been shown that in IPF, S100a4 confers fibrogenicity also on mesenchymal progenitor cells (28), and that its expression in kidney and liver fibrosis coincides with the presence of macrophages. Since lung fibrosis is also driven by monocyte-derived macrophages (29), we hypothesized that macrophage-derived S100a4 might also contribute to the development of lung fibrosis. Here, we present data consistent with this hypothesis: when compared to healthy controls, IHC staining of sections of fibrotic lungs of mice showed increased numbers of S100a4-positive cells which co-localized with Arg1 expressing M2 AMs. S100a4 was found to be strongly upregulated in BALF of two independent murine models of pulmonary fibrosis. Consistent with the findings in mice, we also observed macrophages expressing S100A4 in lung sections of both a control and an IPF patient, and detected S100A4 in BAL fluids of both control and IPF patients. However, we did not observe a difference between the two groups which may be due to the very limited number of samples studied so far. S100A4 was also found to be significantly increased in BAL fluids of COPD patients when compared to controls (30). On the other hand, a recent deep proteome profiling of tissue from human end stage ILD cases and healthy donor controls did not reveal differences in S100A4 protein levels (31). Thus, the role of macrophage-derived S100A4 in human lung fibrosis requires further investigations.

Our *in vitro* studies demonstrated induction of S100a4 in M2-polarized murine primary AMs. S100a4 has both intracellular as well as extracellular functions. Intracellular S100a4 plays a dynamic role in numerous biological processes that are fundamental for cell homeostasis and differentiation (32). When secreted extracellularly, S100a4 serves as a cytokine that regulates cell survival, migration, and differentiation and remodeling of ECM in cancer cells (32). Here, we determined the effects of extracellular S100a4 on pulmonary fibroblasts, which are the most prominent cellular players in the production of ECM, such as collagen and fibronectin, in the lung. We provided the following lines of evidence to demonstrate a profibrotic role of extracellular S100a4 in pulmonary fibrosis: (i) recombinant S100a4 protein had cell growth-promoting properties on lung fibroblasts. (ii) S100a4 induced the expression of the mesenchymal markers α-SMA and collagen 1, indicating that S100a4 promoted lung fibroblasts transition to myofibroblasts which, in turn, synthesized elevated levels of ECM components. Blockage of S100a4 with neutralizing antibodies reduced those effects. Our results are in agreement with the study by Lin et al., reporting an S100a4-induced activation of hepatic stellate cells to promote liver fibrosis (13).

Since the discovery of S100a4, several studies have proven a central role of S100a4 in metastasis formation or liver fibrogenesis. Hence, targeting S100a4 expression provides a promising strategy for rational therapies. Previous research has shown that inhibition of S100a4 expression in cancer cell lines suppressed cell invasion *in vitro* (33). *In vivo* treatment with anti-S100a4-shRNA or S100a4-neutralizing antibody in fibrotic mice reduced accumulation of myofibroblasts, suppressed deposition of collagen, and, therefore, ameliorated the development of liver fibrosis (13). Furthermore, Sack and colleagues performed high-throughput screening of 1,280 pharmacologically active compounds to identify a transcription inhibitor of S100a4 using a human colon cancer cell line. Niclosamide and calcimycin were identified as potential candidates (20, 27). Niclosamide [5-chloro-N-(2-chloro-4-nitrophenyl)-2-hydroxybenzamide] is an FDA-approved anti-helminthic compound used both in humans and animals for the treatment of tapeworm infection since more than 40 years (34). It has been reported that niclosamide inhibited the constitutively active WNT/CTNNB1 signaling pathway by hindering the formation of CTNNB1/TCF transcription activating complex at the S100a4 promoter, thus inhibiting the expression of S100a4 at the transcriptional level. *In vitro* treatment with niclosamide inhibited S100a4-induced migration and proliferation of human colon cancer cells. Besides, niclosamide treatment also attenuated S100a4-induced metastasis formation *in vivo* (20). Calcimycin is one of few natural ionophore antibiotics that specifically transport divalent cations such as calcium and magnesium (35). It has been shown that calcimycin treatment inhibited the constitutively active WNT/β-catenin pathway, thus hindering S100a4 expression and attenuating the S100a4-induced cell migration and invasion both *in vitro* and *in vivo*. Moreover, calcimycin has been reported to reduce the expression of S100a4 at the mRNA level in human monocytes and lymphocytes (36). In line with these findings, both niclosamide and calcimycin were, in our study, able to abrogate the expression of S100a4 during M2 polarization without influencing the polarization of macrophages. Most importantly, niclosamide treatment of bleomycin-instilled mice significantly reduced both the amount of S100a4 in the BAL fluid and the development of lung fibrosis in 50% of the treated mice. The reason why the beneficial effect of niclosamide was observed only in 50% of the treated mice is currently not known. Since it is known that the response to intratracheal bleomycin instillation can be heterogeneous, we cannot completely rule out the possibility that some mice received less bleomycin than others. However, both groups receiving bleomycin displayed a significantly reduced body weight at day 7 after bleomycin instillation (i.e., at the beginning of the niclosamide treatment), when compared to the PBS control groups, suggesting a comparable response to bleomycin (Figure S8 in Supplementary Material). In contrast, the weight loss between the two bleomycin groups was not significantly different, albeit it appeared less pronounced in the niclosamide group. Therefore, we speculate that the niclosamide treatment regimen we have applied was sufficient to ameliorate fibrosis development in response to a certain effective dose of bleomycin but not beyond that. Still, our results suggest that S100a4 warrants further investigation as a therapeutic target in pulmonary fibrosis. Treatment with niclosamide clearly reduced the levels of S100a4 *in vivo* (Figure 7C), yet, it should be noted that niclosamide is not specific for S100a4. In pulmonary fibrosis, this unspecificity may even be favorable, since niclosamide co-inhibits several pathways (e.g., STAT3, AKT, and Wnt/β-catenin) which have been shown to contribute to fibrosis development. Consistent with that are recent findings demonstrating prevention of systemic sclerosis by niclosamide treatment in a reactive oxygen species-induced mouse model (37). It should be noted, however, that so far all data including ours are derived from well established mouse models, and that the relevance for human fibrosis still remains to be shown.

## Ethics Statement

All animal experiments were in compliance with the German Animal Welfare Act (German Federal Law §8 Abs. 1 TierSchG), and the protocols were approved by the local Animal Care and Use Committee (District Government of Upper Bavaria; permit number 124-08, 154-13, and 130-14). Human lung tissue was obtained from the Comprehensive Pneumology Center cohort of the BioArchive CPC-M at the University Hospital Grosshadern of the Ludwig Maximilian University. Participants provided written informed consent to participate in this study, in accordance with approval by the local ethics committee of the LMU, Germany (Project 333-10, 455-12).

## Author Contributions

HA, TS, and MK conceived and designed the research. WZ, SO, BS, SK, CS-W, DW, ML, and HA planned and performed the experiments and analyzed the data. HA, WZ, and MK wrote the manuscript, and all authors approved the final version of the manuscript.

## Conflict of Interest Statement

The authors declare that the research was conducted in the absence of any commercial or financial relationships that could be construed as a potential conflict of interest. The reviewer MK and handling Editor declared their shared affiliation.
