# Supplementary material for: S100a4 Is Secreted by Alternatively Activated Alveolar Macrophages and Promotes Activation of Lung Fibroblasts in Pulmonary Fibrosis
Source: Front Immunol. 2018 Jun 1;9:1216. doi: 10.3389/fimmu.2018.01216 (PMC5992816; doi:10.3389/fimmu.2018.01216)
Supplement: Supplementary file 1 [file presentation_1.PDF]

Supplementary Material

S100a4 Secreted by Alternatively Activated Alveolar Macrophages Promotes Activation of Lung Fibroblasts in Pulmonary Fibrosis

Wei Zhang [1], Shinji Ohno [1#], Beatrix Steer [1], Stephan Klee [1], Claudia A. Staab-Weijnitz [2], Darcy Wagner [1#], Mareike Lehmann [1], Tobias Stoeger [2], Melanie Königshoff [1, 3], and Heiko Adler [1*]

[1] Comprehensive Pneumology Center, Research Unit Lung Repair and Regeneration, Helmholtz Zentrum München - German Research Center for Environmental Health (GmbH), Marchioninistrasse 25, D-81377 Munich, Germany, and University Hospital Grosshadern, Ludwig-Maximilians-University, Munich, Germany; Member of the German Center of Lung Research (DZL)

[2] Helmholtz Zentrum München - German Research Center for Environmental Health (GmbH), Institute of Lung Biology and Disease, Comprehensive Pneumology Center, Ingolstädter Landstr. 1, D-85764 Neuherberg, Germany

[3] Division of Pulmonary Sciences and Critical Care Medicine, Department of Medicine, University of Colorado Denver, Aurora, CO, USA.

# Current address: S.O.: Department of Virology, Graduate School of Medicine, University of the Ryukyus, 207 Uehara, Nishihara, Nakagami, Okinawa, 903-2015, Japan; D.W.: Lung Bioengineering and Regeneration, Lund University, Box 117, 221 00 LUND, Sweden

Supplementary Figures

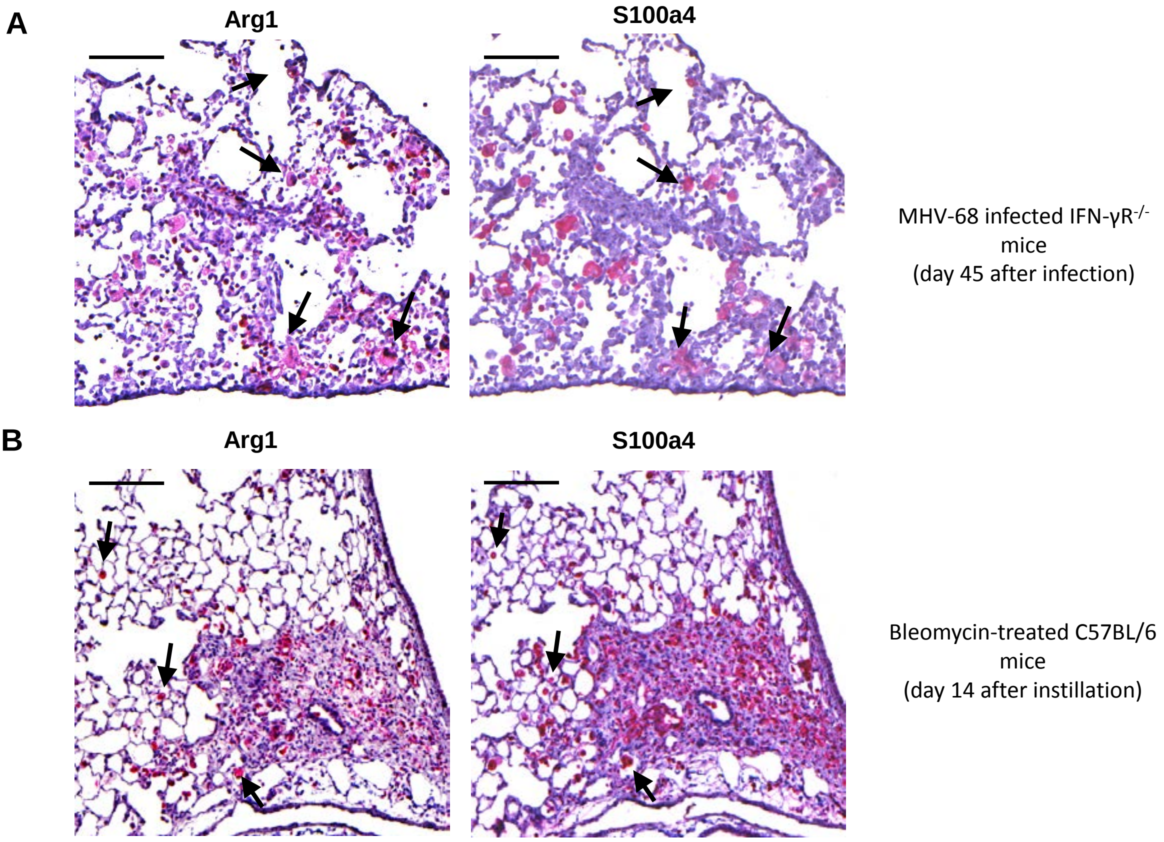

Supplementary Figure S1: Co-localization of S100a4 with alternatively activated macrophages in tissue sections from fibrotic mice. A) Serial section staining of S100a4 and Arg1 (M2 macrophages) on lung serial sections of MHV-68 infected IFN-γR$^{-/-}$ mice at day 45 after infection. Arrows indicate co-staining. B) Serial section staining of S100a4 and Arg1 (M2 macrophages) on lung serial sections of bleomycin-treated mice at day 14 after instillation. Arrows indicate co-staining. Scale bar: 100 µm.

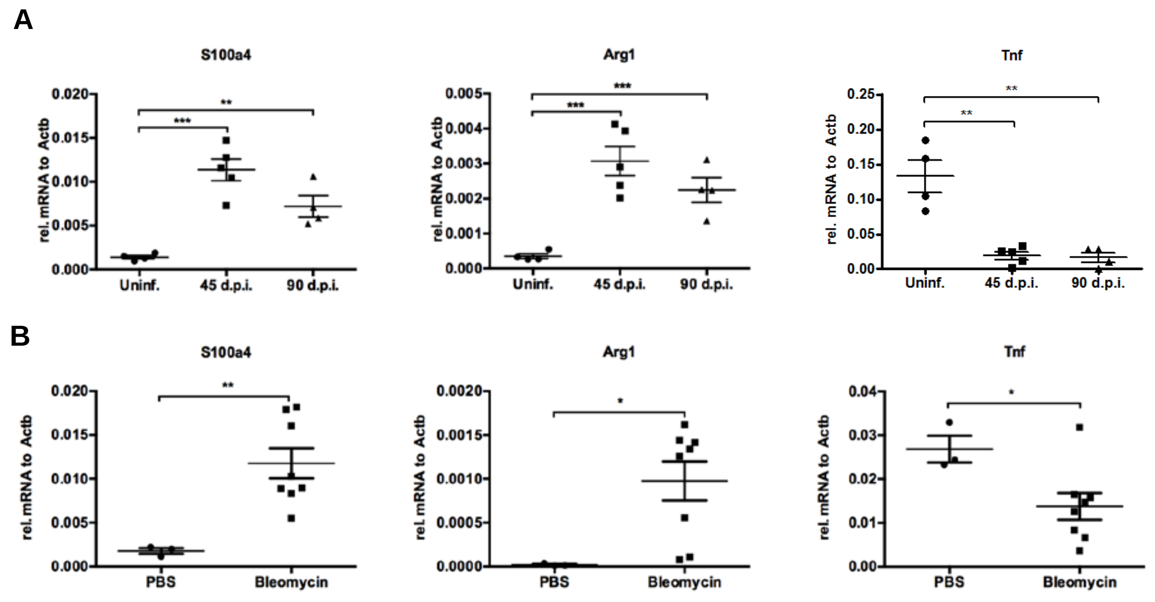

Supplementary Figure S2: Increased S100a4 expression in alveolar macrophages in experimental lung fibrosis. A) qRT-PCR was used to determine the levels of S100a4, Arg1 (M2 marker) and TNF-α (M1 marker) transcripts in alveolar macrophages of uninfected and MHV-68 infected IFN-γR$^{-/-}$ mice at days 45 and 90 after infection. Expression of target genes was normalized to β-actin. Each symbol represents a mouse (n = 4 uninfected group; n = 5 or 4 infected group). Results are shown as mean ± SD. Unpaired t-test was performed for statistical analysis (** denotes $p < 0.01$; ***denotes $p < 0.001$). (B) Relative mRNA levels of S100a4, Arg1 and TNF-α in alveolar macrophages isolated from PBS or bleomycin challenged C57BL/6 mice were assessed. Expression of target genes was normalized to β-actin. Each symbol represents a mouse (n = 3 PBS group; n = 8 bleomycin-treated group). Results are shown as mean ± SD. Unpaired t-test was performed for statistical analysis (* denotes $p < 0.05$; ** denotes $p < 0.01$).

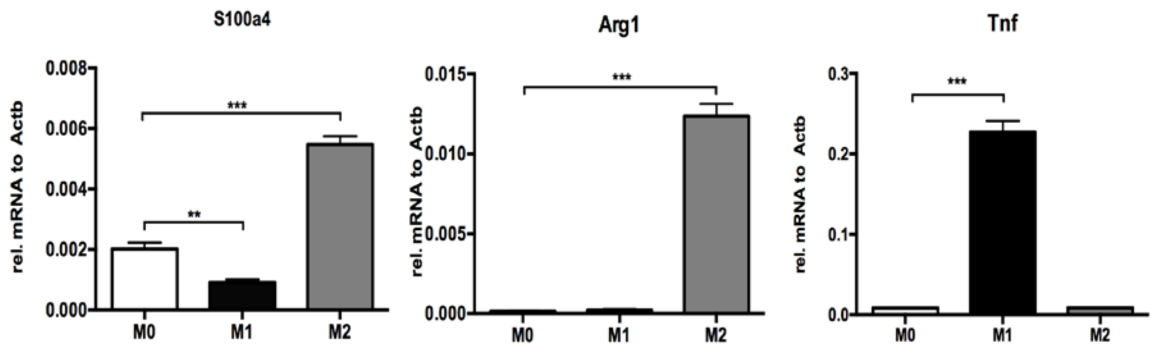

Supplementary Figure S3: Expression profile of S100a4 in polarized alveolar macrophages. Alveolar macrophages isolated from C57BL/6 mice were treated with LPS (1 μg/ml) and IFNγ (20 ng/ml) or IL-4 (20 ng/ml) for 24 hours and relative mRNA levels of S100a4, Arg1 (M2 marker) and TNF-α (M1 marker) were assessed using qRT-PCR. Expression of target genes was normalized to β-actin. qRT-PCR results are representative of two independent experiments with similar results. Shown are mean ± SD of triplicate samples from one of two representative experiments. Unpaired t-test was performed for statistical analysis (** denotes $p < 0.01$; ***denotes $p < 0.001$).

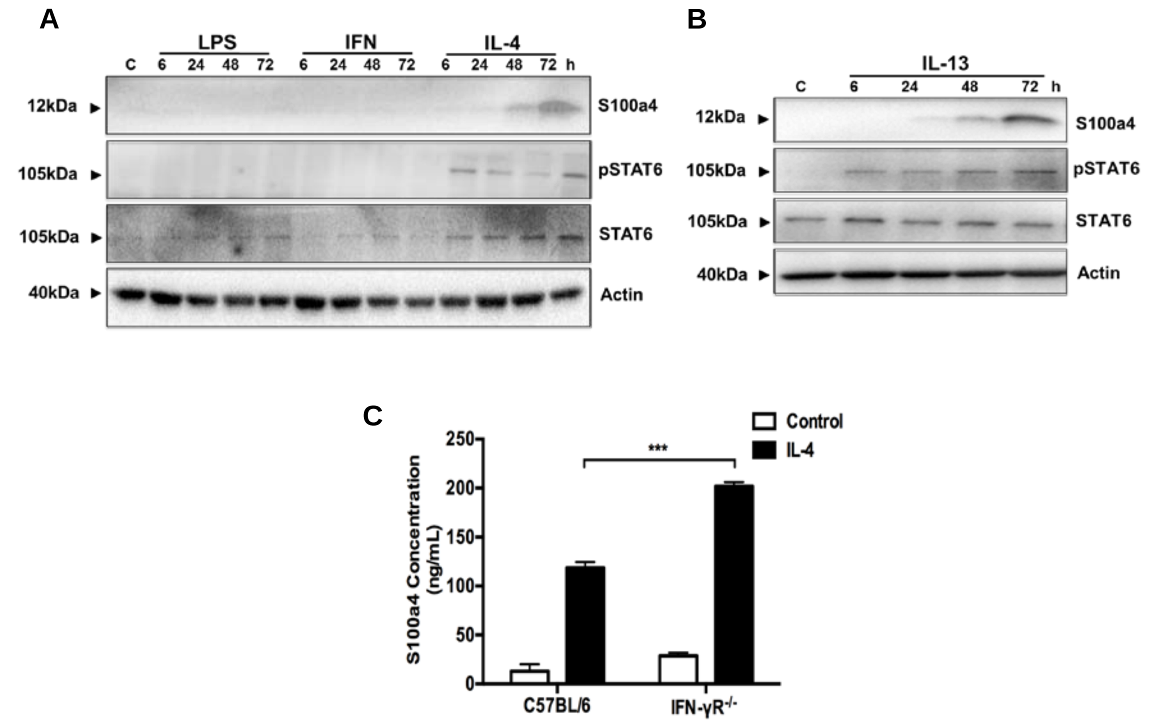

Supplementary Figure S4: Analysis of S100a4 protein expression during alveolar macrophage polarization. A) Primary alveolar macrophages isolated from C57BL/6 mice were treated with LPS (1 μg/ml), IFNγ (20 ng/ml), IL-4 (20 ng/ml) or B) with IL-13 (20 ng/ml) for 6, 24, 48 and 72 hours, respectively. Protein expression of S100a4 and phosphorylation of STAT6 were determined by western blot assay. The phosphorylated STAT6 was used as an indicator for IL-4 or IL-13 induced M2 macrophage polarization. Blots were incubated with an anti-β-actin antibody as loading control. Results are representative of two independent experiments. The control (c) reflects unstimulated alveolar macrophages at 24 hours. C) Primary alveolar macrophages from IFN-γR$^{-/-}$ and C57BL/6 mice were treated with IL-4 (20 ng/ml) for 72 hours. The supernatant was collected for the ELISA assay. Results are representative of two independent experiments with similar results. Shown are mean ± SD of triplicate samples from one experiment. Unpaired t-test was performed for statistical analysis (***denotes $p < 0.001$).

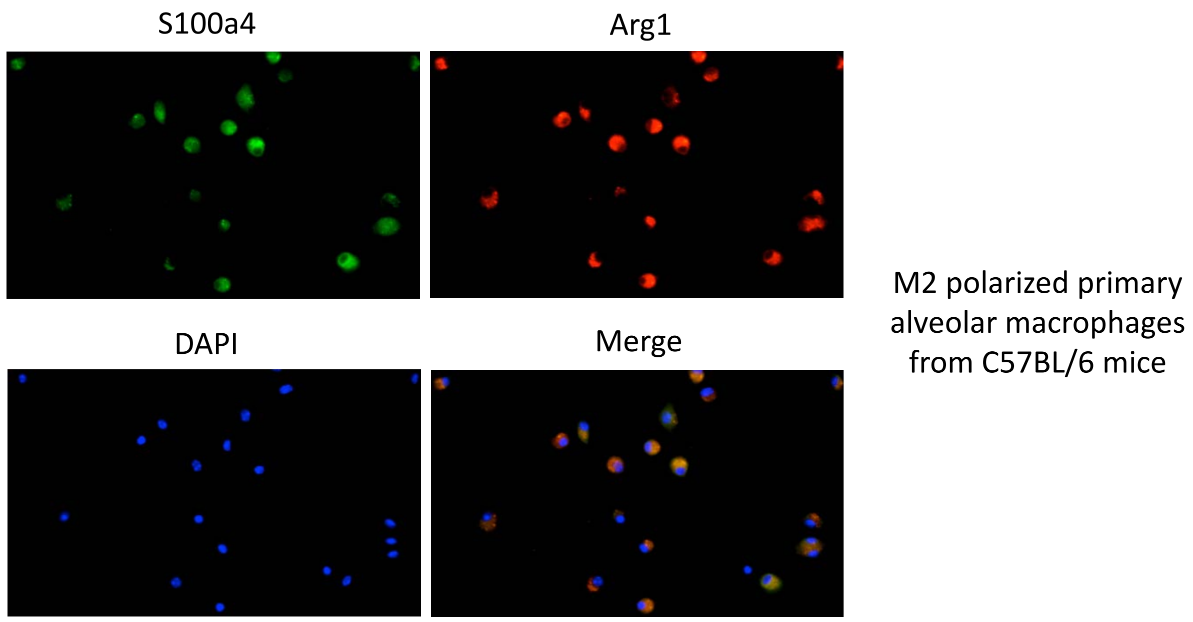

Supplementary Figure S5: S100a4 co-localizes with Arg1 in M2 polarized alveolar macrophages. Primary alveolar macrophages from C57BL/6 mice were seeded on coverslips and polarized with IL-4 (20 ng/ml) for 48 hours. Green and red fluorescences reflect S100a4 and Arg1, respectively. Yellow indicates co-localization of the two proteins, whereas DAPI depicts nuclei. Results are representative of three independent experiments.

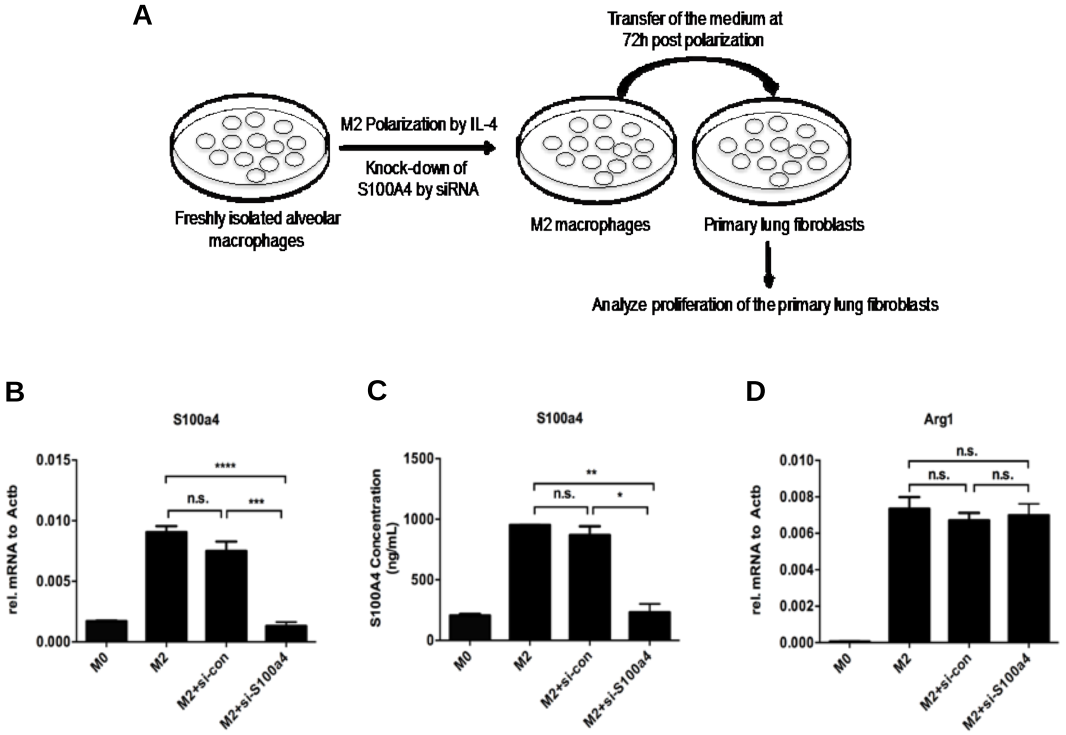

Supplementary Figure S6: Investigation of the effect of endogenous S100a4 produced by M2 macrophages on primary lung fibroblasts. A) Schematic presentation of the experiment. A total of 3×10^5 freshly isolated alveolar macrophages, isolated from C57BL/6 mice, were plated in a 24-well plate, polarized by IL-4 and simultaneously transfected with anti-S100a4 siRNA or scrambled control siRNA. The supernatant (conditioned medium) was collected after 72 hours and transferred to primary lung fibroblasts. The proliferation of the primary lung fibroblasts was subsequently analyzed. B) and C) M2 polarized alveolar macrophages were transfected with nonspecific scrambled control siRNA or S100a4-specific siRNA for 72 hours, and the levels of S100a4 were measured to examine knockdown efficiency by RT-PCR (B) and ELISA (C). D) RT-PCR was performed for Arg1 mRNA expression analyses. Results are representative of two independent experiments with similar results. Shown are mean ± SD of triplicate samples from one experiment. Unpaired t-test was performed for statistical analysis (* denotes $p < 0.05$; ** denotes $p < 0.01$; *** denotes $p < 0.001$; **** denotes $p < 0.0001$; n.s. denotes non-significance).

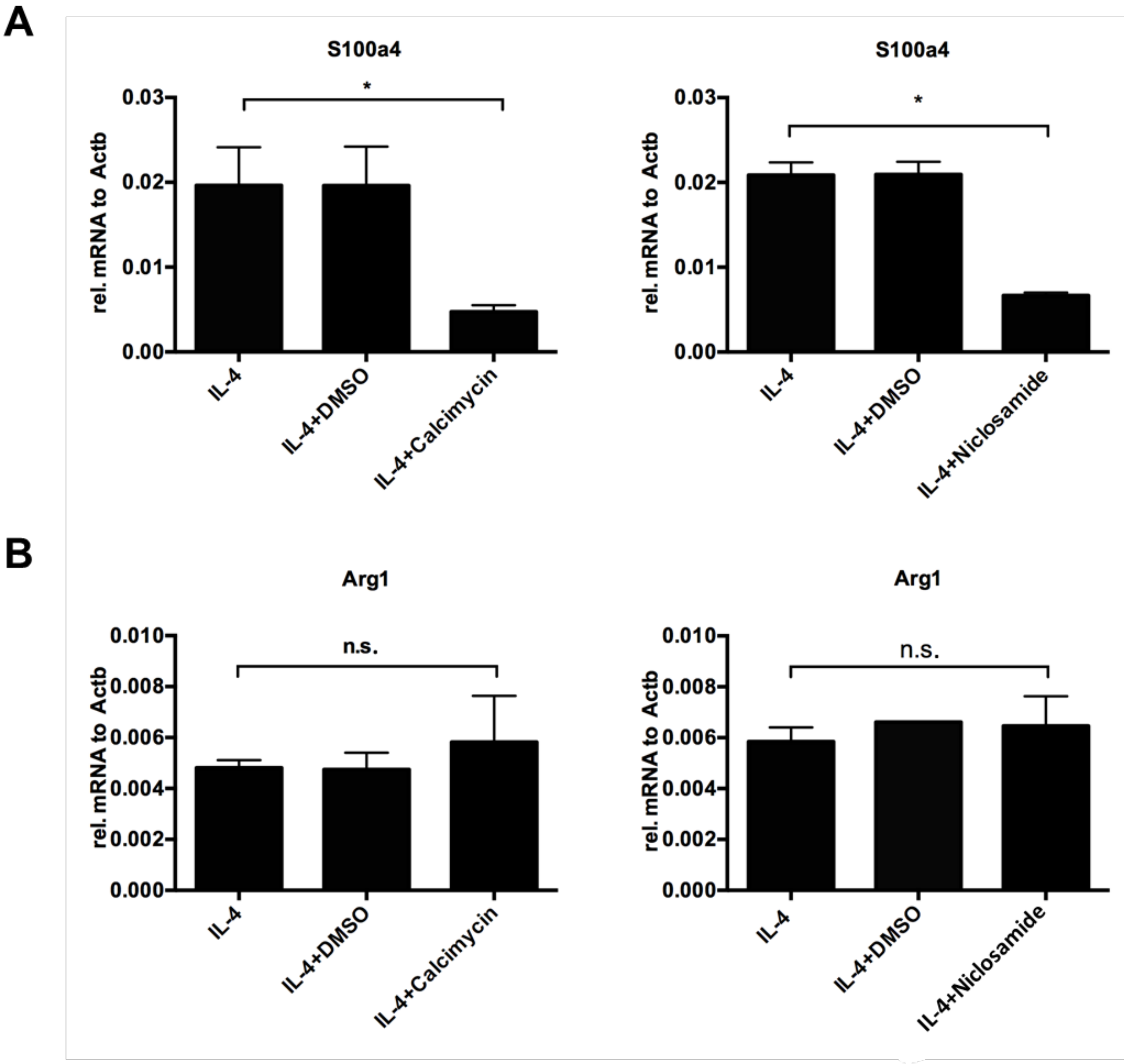

Supplementary Figure S7: Calcimycin and niclosamide inhibit expression of S100a4 during M2 polarization. Alveolar macrophages were treated with IL-4 (20 ng/ml) and 1 μM calcimycin or 0.3 μM niclosamide, respectively, for 24 hours. mRNA levels of S100a4 (A) and Arg1 (B) were determined by qRT-PCR. Results are normalized to *β-actin* expression. Results are representative of two independent experiments with similar results. Shown are mean ± SD of duplicates or triplicates from one experiment. (* denotes $p < 0.05$; n.s. denotes non-significance).

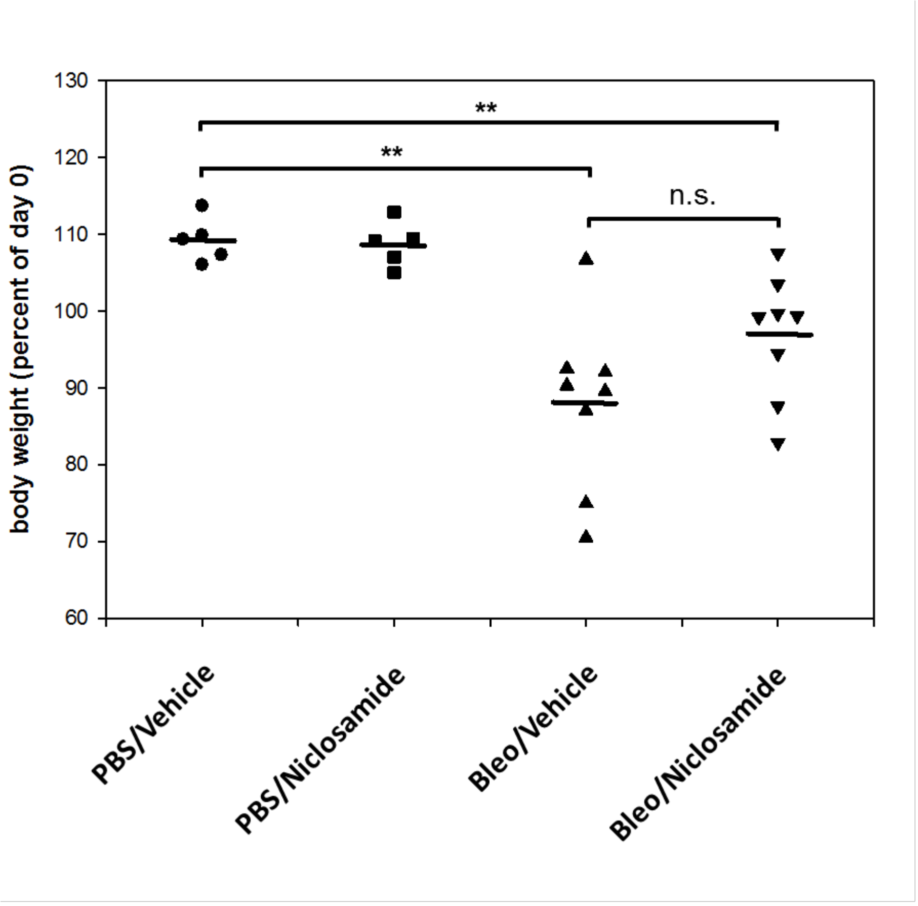

Supplementary Figure S8: Body weight of mice at the beginning of niclosamide treatment (day 7 after bleomycin instillation). Mice were intratracheally treated with bleomycin, or as a control, with PBS. Each symbol represents the body weight of an individual mouse (as percent of day 0), and the bars represent the mean. Unpaired t-test was performed for statistical analysis (** denotes $p < 0.01$; n.s. denotes non-significance).
